# Hydration status during commercial saturation diving measured by bioimpedance and urine specific gravity

**DOI:** 10.3389/fphys.2022.971757

**Published:** 2022-09-29

**Authors:** Stian Lande Wekre, Halvor Dagssøn Landsverk, Jacky Lautridou, Astrid Hjelde, Jean Pierre Imbert, Costantino Balestra, Ingrid Eftedal

**Affiliations:** ^1^ Department of Circulation and Medical Imaging, Faculty of Medicine and Health Sciences, NTNU Norwegian University of Science and Technology, Trondheim, Norway; ^2^ Divetech, Biot, France; ^3^ Environmental and Occupational Physiology Laboratory, Haute Ecole Bruxelles-Brabant HE2B, Brussels, Belgium; ^4^ DAN Europe Research, Brussels, Belgium

**Keywords:** bioimpedance (BIA), hydration, hyperbaric saturation, saturation diving, total body water, underwater work, decompression

## Abstract

Excessive fluid loss triggered by hyperbaric pressure, water immersion and hot water suits causes saturation divers to be at risk of dehydration. Dehydration is associated with reductions in mental and physical performance, resulting in less effective work and an increased risk of work-related accidents. In this study we examined the hydration status of 11 male divers over 19 days of a commercial saturation diving campaign to a working depth of 74 m, using two non-invasive methods: Bioelectrical impedance analysis (BIA) and urine specific gravity (USG). Measurements were made daily before and after bell runs, and the BIA data was used to calculated total body water (TBW). We found that BIA and USG were weakly negatively correlated, probably reflecting differences in what they measure. TBW was significantly increased after bell runs for all divers, but more so for bellmen than for in-water divers. There were no progressing changes in TBW over the 19-day study period, indicating that the divers’ routines were sufficient for maintaining their hydration levels on short and long term.

## Introduction

Commercial saturation divers must control their fluid intake to compensate for losses triggered by their hyperbaric work environment. Failure to do so leads to dehydration with negative effects on cognitive and physical performance. As the divers cannot drink while they are in the water, attention is required during the time they spend in the dive bell during underwater work excursions.

A water deficit of 2% or more of the body weight impairs mental performance in the areas of short-term memory, arithmetic efficiency, and visuomotor tracking (Gopinathan et al., 1988) as well as aerobic work performance (Erdman and Appel, 2005). Also, dehydrated individuals report higher levels of perceived effort, reduced alertness and increased levels of tiredness and fatigue (Cian et al., 2000; Szinnai et al., 2005). It is known that dehydrated workers are less effective and at increased risk of accidents (Kenefick and Sawka, 2007). For this reason, the United Kingdom Health and Safety Executive recommends 100–200 ml of water every 15 min, and 500 ml of water for every hour of work to be drunk before working in areas where provision of water is impossible (Powell and Bethea, 2005). However, these recommendations are aimed at high heat working environments and do not take the complex physiology in diving into account.

Saturation divers are exposed to multiple factors that contribute to dehydration. The hyperbaric conditions themselves cause a hyperbaric diuresis, also without water immersion (Shiraki et al., 1985). Increased urinary neopterin and creatinine during and immediately after saturation diving have been reported, suggesting a reduction of renal function (Mrakic-Sposta et al., 2020). Water immersion increases the excretion of atrial natriuretic peptide and suppresses vasopressin, thereby increasing the diuresis and excretion of sodium (Miyamoto et al., 1991; Epstein, 1992; Norsk et al., 1993). Saturation divers also use hot water suits to maintain their body temperature in the cold water, and these suits circulate hot seawater in direct contact with the diver’s skin. The thermal stress from the hot water causes additional fluid loss through sweat (Hope et al., 2005), possibly amplified by an osmotic effect of salt water (Hope et al., 2001). Losses of up to 4–5kg, or 5–6% of total body weight, have been reported (Hope, 1995). This water must be replenished. In commercial saturation diving, standards such as the Norwegian NORSOK U-100 include routines for fluid intake (Norge Standard, 2015).

Hydration status can be measured in several ways. Doing it in the demanding environment of saturation diving poses specific challenges, including high ambient pressure, restricted use of electrical devices, and infection risk from invasive procedures. Bioelectrical impedance analysis (BIA) provides an easy to use, low cost, and non-invasive method (O’brien et al., 2002). BIA measurements can be made by the divers themselves inside the pressurized living chambers. Urine specific gravity (USG) is also widely used as a measure of hydration (Kavouras, 2002), and urine samples are easily obtainable. USG can be measured by a refractometer outside the pressurized living chambers.

In this study we examined divers’ hydration status during the bottom phase of a commercial saturation dive to determine whether they were successful at compensating for dehydration. Two different methods, BIA and USG, were used to before and after daily bell runs for underwater work excursions, to determine whether there were acute and/or progressing changes in total body water (TBW) during 18 days of saturation diving.

## Materials and methods

### Ethics

The study protocol was approved in advance by the Norwegian Regional Committee for Medical and Health Research Ethics (REK), approval reference ID 117404. All eligible subjects were provided with information regarding the aim and scope of the study, in addition to experimental procedures and data handling. Written consent was given before inclusion. The experimental procedures were conducted according to the Declaration of Helsinki principles for ethical human experimentation.

### Study subjects

The study subjects consisted of 11 male saturation divers who passed a pre-dive medical examination before they were committed to saturation diving on board TechnipFMC’s dive support vessel (DSV) Deep Arctic. Select anthropometric data for the subjects are shown in Table 1.

**TABLE 1:** Anthropometric data for the 11 study subjects.

	Age (years)	Height (cm)	Weight (kg)	BMI (kg/m^2^)
Min	33	169	72	20.8
Max	67	196	115	31.2
Mean (±SD)	46.2 ± 11.6	184.6 ± 8.6	93.6 ± 17.0	27.2 ± 3.2

Mean given as arithmetic means ± standard deviation.

### Saturation diving

**FIGURE 1:**
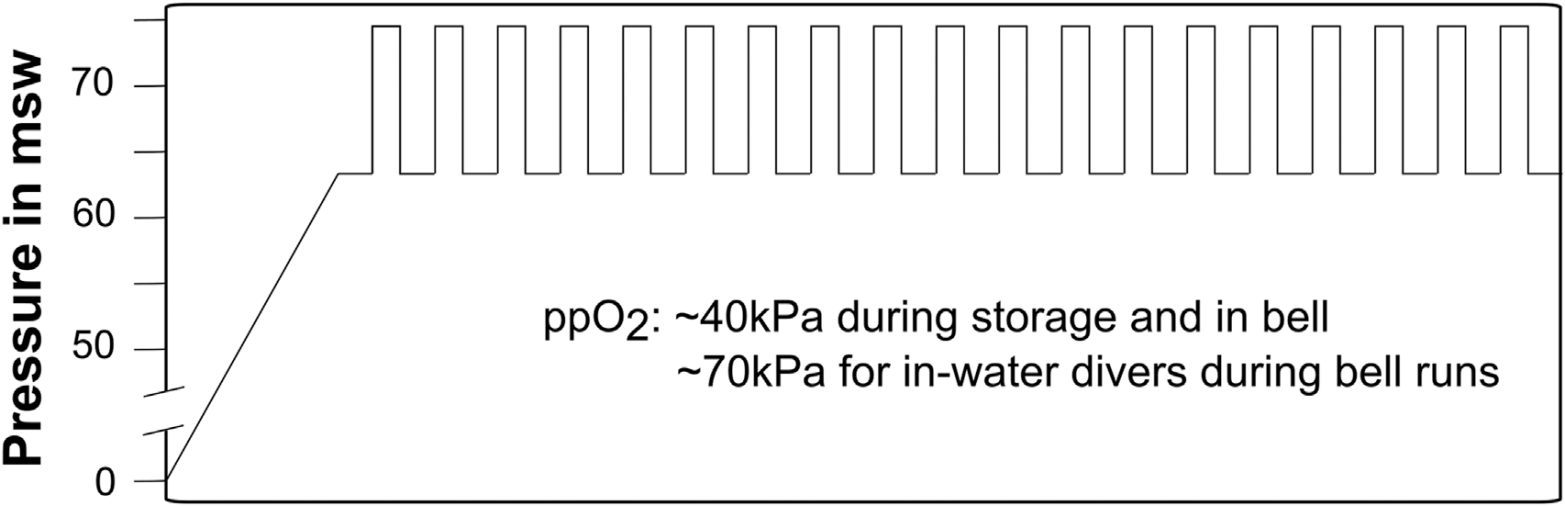
Heliox saturation dive profile. Vertical bars indicate bell runs. The storage depth was kept at 63 msw, and the maximum working depth was 74 msw. Partial pressure of oxygen was kept close to 40 kPa during storage, and raised during bell runs to 60–80 kPa for the in-water divers and 40–50 kPa for the bellmen.

The study was done during a commercial saturation diving campaign on the United Kingdom continental shelf in the summer of 2021. The divers were grouped into four teams, each team consisting of three divers. They were pressurized in heliox to a living depth of 63 m of seawater (msw). The living chambers were kept at 63 msw throughout the study period, with an oxygen pressure of ≈ 40 kPa and temperature controlled around 28–30°C. Humidity in the living chambers was set to 40%–50%. The teams were on 12-h shifts, with a new team starting their shift every 6 hours. Each team worked the same shift for the entire campaign, and thus had to adapt to different circadian rhythms. During shifts, the divers would start with personal morning routines (breakfast, hygiene etc.) before continuing with a brief of the day’s planned work. They would then proceed to prepare their equipment and move into one of the vessel’s two diving bells. The bell was sealed and lowered into a moon pool to deploy the divers to the ocean floor. Once at bottom, two divers would go leave the bell for in-water work, while the third– the bellman - remained in the bell. The role of bellman and in-water diver rotated so that each diver took his turn as bellman every third day. The bell oxygen was controlled at around 40–50 kPa. The diving mix provided a ppO2 ranging from 60 to 80 kPa. One bell run lasted up to 8 h, with a maximum of 6 h of in-water work. A new team would be heading down while the previous team was going up, ensuring continuous underwater activity. The saturation diving profile is illustrated in Figure 1.

### Hydration routines

During bell runs, the divers had free access to bottled drinking water, coffee, tea, and fruit. They had sport tablets (GO Hydro electrolyte tablets, Science in sport, London, United Kingdom) to add to the water if they preferred isotonic drink. The divers where not monitored for daily intake of sport tablets during this study; however, a survey conducted pre- and post-saturation showed that over half of the divers on this vessel used them. They took 1-4 tablets a day when they were in-water divers and none on the days they were bellman. Hydration routines during bell runs were per the NORSOK U-100 standards (Norge Standard, 2015), which requires in-water divers to return to the bell and take their helmet off for at least 30 min for rehydration and lunch during the third or fourth working hour. In-water divers were also at liberty to return to the bell to drink at any time at their own request. The bellman always had access to bottled drinking water and fruit.

### Data and urine collection

Bioimpedance (BIA) data and urine were collected by the study subjects themselves. After enrollment into the study, they received training in use of the BIA device, Biody Xpert ZM from Aminogram (La Ciotat, France), as per the manufacturer’s instructions. Conduction between the skin and the electrodes was standardized by wetting with a small amount of electrode gel (Spectra 360 electrode gel, Parker Laboratories, Fairfield, NJ, United States). The device is a multifrequency impedance meter, accredited to the ISO 13485:2016 standard and CE marked, measuring impedance at 200KHz, 100KHz, 50Khz, 20 KHz and 5 KHz in addition to the phase angle at 50 KHz. It is handheld and operated by a single 9V PP3 battery, and suitable to operate within the pressurized living chambers. Data was transferred over Bluetooth to a smartphone outside the chambers, using the proprietary Aminogram Biodymanager-app for android. The data is directly transferred from the device, without interaction from the divers, ensuring truthful data. Urine was collected into 50 ml containers as close to the bioimpedance measurements as feasible, decompressed *via* the medical lock, and analyzed in the DSV hospital using a digital refractometer (ORF-P, KERN & SOHN GmbH, Balingen, Germany).

BIA and USG were first taken after the pre-dive medical examinations before the divers entered the pressure chambers to be compressed (baseline), and then daily before and after bell runs. The divers were instructed to collect urine and measure bioimpedance before breakfast but after drinking fluids, and again as soon as possible after the bell runs, before their next meal. The procedure was repeated daily during 19 days of diving and ended before the decompression back to the surface. Bell runs were conducted continuously during the bottom phase, except for 2 days where the ship moved to shore for crew shifts. This resulted in data and samples from 18 bell runs for one team, and 19 for the three others.

### Total body water calculation

Total body water (TBW) was calculated from the bioimpedance data using the equation by Kushner and Schoeller (1986), as recommended by Aminogram.

$$TBW=\left(0.382+\left(0.014*Sex\right)\right)*\left(\frac{H^{2}}{Z_{50kHz}}\right)+\left(0.105+0.038\right)*W+0.084*Sex.$$ (1)

TBW = Total body water in liters, Z_50kHz_ = impedance at 50 kHz, Sex = 1 for males and 0 for females, H = height in cm, W = weight in kg.

### Statistics

The following questions were asked in the statistical analysis: 1) Were USG and BIA correlated? 2) Was there a difference in hydration between in-water divers and bellmen after a single bell run? And 3) Was there a progressing change in hydration over time spent in saturation? For this final question, the data were pooled into three batches for comparison: days 1–6, days 7–12 and days 13–19. Statistical significance was set *a priori* to *p* < 0.05 for all tests.

Statistical analysis was done in GraphPad Prism version 9.3.1 for Mac (GraphPad Software, San Diego, CA, United States) or IBM SPSS Statistics for Windows (Version 27.0. Armonk, NY: IBM Corp). Prior to the analysis, the data were checked for normality by Shapiro-Wilk’s test and visual inspection of Q-Q plots. Normally distributed data were analyzed by parametric tests, whereas data that were not normally distributed were analyzed using non-parametric tests.

BIA and USG data were compared using the Spearman rank correlation coefficient. Days with missing data points were excluded, resulting in an equal sample size for all calculations in the correlation matrix (*n* = 174). Absolute values BIA at 50 kHz and USG were used for the comparison. Differences between in-water diver and bellman data were analyzed using a paired t-test for normally distributed data, and a Wilcoxon matched pairs test for data that were not normally distributed. To analyze changes in hydration over time we applied a repeated measures analysis of variance (ANOVA). Post hoc comparisons using Tukey’s test were performed where a significant difference was indicated by the ANOVA. Mauchly’s test of sphericity was used to validate the ANOVA, and the Greenhouse & Geisser correction was applied where the assumption of sphericity was not met.

## Results

### Correlation between bioelectrical impedance analysis and urine specific gravity

**FIGURE 2:**
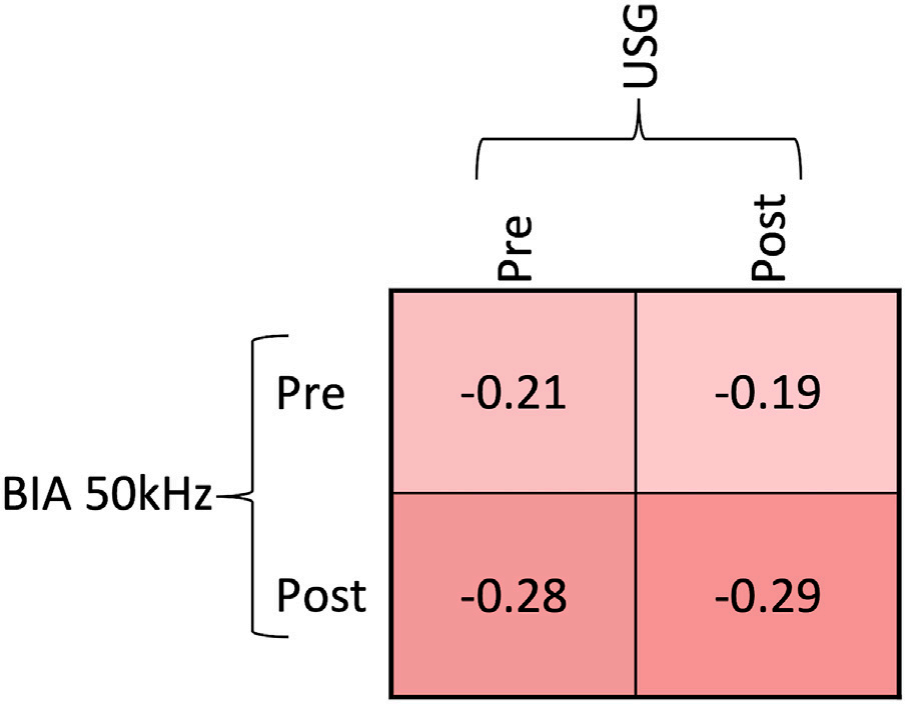
The relationship between pre- and post-bell run values of USG and BIA at 50 kHz analyzed by Spearman correlation coefficients. All coefficients were statistically significant (*p* < 0,02), implying that there was a negative correlation between USG and BIA.

BIA and USG showed a weak but significant negative correlation. As BIA measurements are inversely proportional to TBW, this was the opposite of what was expected and implies that the divers’ body water was higher at time points when their urine was denser. The results of the correlation analysis are shown in Figure 2.

### Comparison of in-water divers and bellmen after single bell runs

**FIGURE 3:**
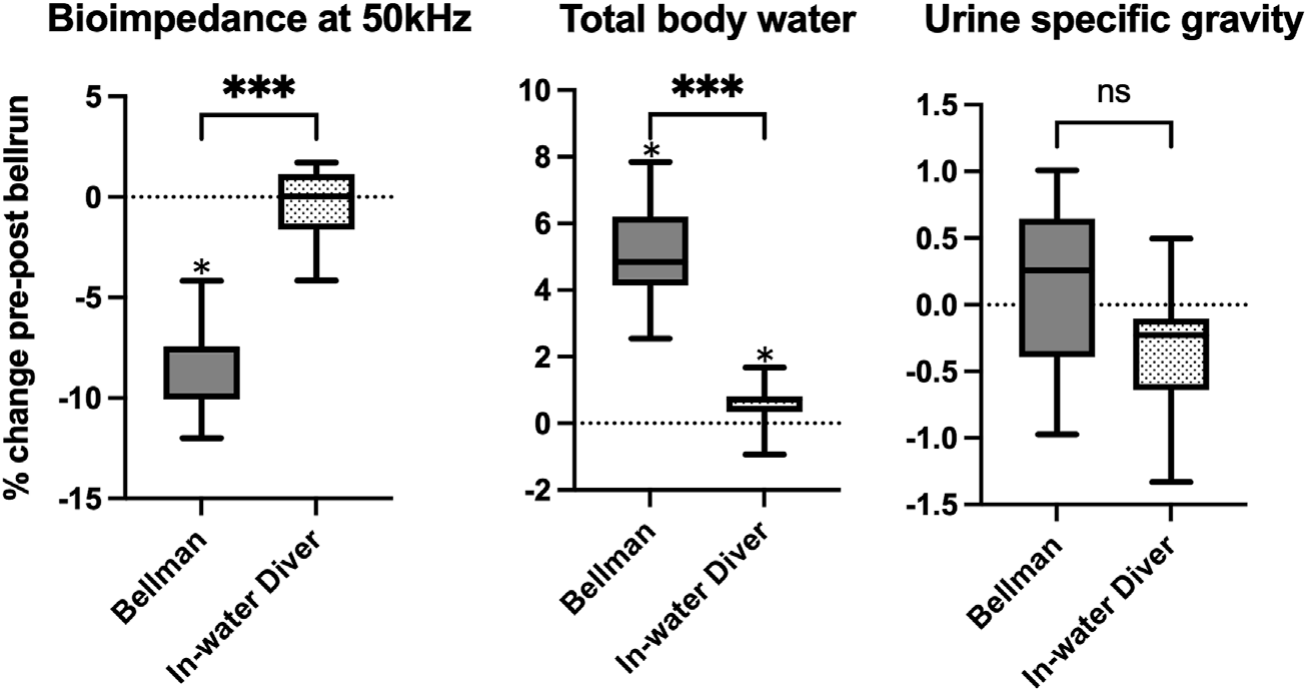
Boxplot showing the % change in hydration markers during bell runs, for bellmen and in-water divers. Box edges represent 25th and 75th percentiles. Whiskers are minimum and maximum values. Calculation of differences were done using a paired t-test for urine specific gravity and Wilcoxon matched pairs test for bioimpedance and total body water. *median different from zero (*p* ≤ 0,05). *** significant difference between bellmen and in-water divers (*p* ≤ 0,001). “ns” no significant difference between bellmen and in-water divers.

BIA was significantly lower after a single bell run for the bellmen, whereas no change was seen for the in-water divers. By inference, TBW increased after bell runs for the bellmen. However, the TBW calculation exposed a statistically significant increase also for the in-water divers: the Wilcoxon matched pairs test showed that the bellmen had a median change in total body water of 4.8 percent, whereas the in-water divers had a median change of 0.7 percent, giving a median of differences of 4.1 (*p* = 0.001). There were no changes in USG after bell runs. Results of single bell run comparisons are shown in Figure 3.

### Total body water during time spent in hyperbaric saturation

**FIGURE 4:**
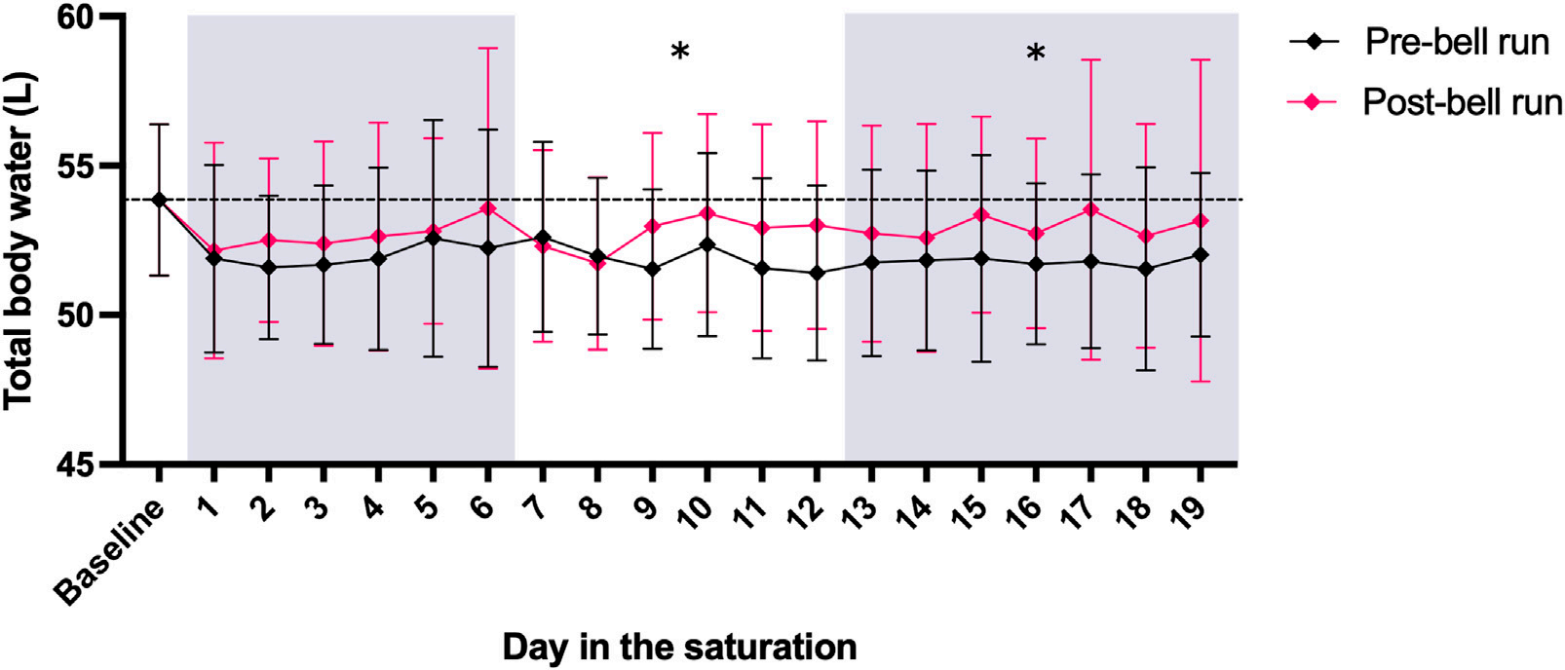
TBW calculated from BIA data collected pre- and post-bell runs from 11 saturation divers Diamonds and whiskers are means ± SD. The mean pre-saturation TBW baseline is shown as a horizontal stapled line. Panel shading indicates the three periods for which data were pooled to analyze progressing changes in TBW. “*” indicates that the mean of pre-bell run TBW for the period was significantly below the pre-saturation baseline. There were no differences in post-bell run TBW compared to baseline (*p* < 0.05).

The divers’ TBW was higher at baseline than during their time in hyperbaric saturation, with differences reaching significance for the two last periods of pooled data: between baseline and days 7–12 (*p* < 0.03, decrease of 3.4%) and days 13–19 (*p* < 0.02, decrease 3.5%). The TBW data are shown in Figure 4.

## Discussion

Dehydration increases the risk of accidents at work. Saturation divers are especially prone to dehydration and must amend their fluid intake to compensate for this. In this study we monitored divers’ hydration status daily during the bottom phase of a commercial saturation diving camping, using two non-invasive methods: BIA and USG. Our main findings were that the divers were not dehydrated after single bell runs, nor did they become progressively dehydrated as the diving campaign proceeded.

Water immersion and elevated environmental pressure both trigger diuresis, and excess fluid loss is common in diving (Hong et al., 1977; Shiraki et al., 1985; Shiraki et al., 1987; Sagawa et al., 1990). A recent study suggests that increased pressure may cause a reduction in renal function (Mrakic-Sposta et al., 2020). In addition, saturation divers suffer increased loss of water through sweating in their hot water suits (Hope et al., 2005), reported to be up to 4–5 kg during a single dive (Hope, 1995). Failure to replenish the lost body water will lead to a state of hypohydration, which is associated with multiple adverse effects on both cognitive (Grandjean and Grandjean, 2007) and physical performance (Barr, 1999). For divers specifically, dehydration has been shown to increase the risk of severe decompression sickness in an animal model of simulated diving (Fahlman and Dromsky, 2006) and pre-dive hydration lowers the risk for decompression sickness (Gempp et al., 2009). The task at hand for saturation divers, such as welding, construction or inspection requires physical fitness and mental alertness. This is further complicated by the diving- and life-support systems for which the requirements are “much stricter than those for life-support systems used in outer space operations” (Brubakk et al., 2014). It is safe to say that dehydration may have negative effects on both the efficiency and safety of a diving operation, and that good hydration routines are essential. We found no progressing dehydration among the divers in our study. Although TBW was higher at baseline than during hyperbaric saturation, no further changes were observed over their time in saturation (Figure 4). Our impression during the study was that both the divers and supporting personnel paid a great deal of attention to the divers’ fluid intake, and the BIA and USG data indicate that their routines were adequate to maintain daily as well as long-term hydration levels.

The significant difference between the bellman and in-water divers must be seen in context of their different exposures. The in-water divers are exposed to effects of immersion and hot water suits and are not at liberty to drink at their own leisure without returning to the bell. The bellman, on the other hand, always has drink available is not affected by neither immersion nor the heat from the suits. The increase in TBW that we saw among the bellmen may very well be because of maintenance of good hydration routines based on their days of in-water diving.

Opposite to what we expected, the BIA and USG data were negatively correlated (Figure 2). This may be explained by differences in what the two methods measure. Both methods are widely used as indices of hydration status, as they are non-invasive, inexpensive, and can be done with minimal equipment. While USG is considered a good measure of hydration status as the concentration of solutes in urine increases with water deficit (Oppliger et al., 2005) and USG is strongly correlated with urine osmolality (Armstrong et al., 1994; Popowski et al., 2001), its correlation decreases in pathological urine (Imran et al., 2010). BIA on the other hand, measures the body’s conducting properties (Kyle et al., 2004). By combining the impedance with the subject’s height and weight it is possible to calculate TBW (Kushner and Schoeller, 1986). However, the BIA method also has its own set of constraints that may influence the results (González-Correa and Caicedo-Eraso, 2012), where consistent limb positioning is the most important (Kushner et al., 1996). The study subjects were given training in BIA, but we cannot eliminate the possibility of variations in body positioning, contact with conducting material, skin-electrode contact etc. When interpreting the BIA data. Also, most equations to calculate TBW are based on euhydrated people, and it has been found that the concomitant fluid and electrolyte changes that occur with dehydration may confound BIA measurements (O’brien et al., 2002). The negative correlation observed between the two methods in our study may also be related to hyperbaric diuresis during exposure to high pressure (Shiraki et al., 1985). This is characterized by an increase in urine flow and decrease in osmolality, associated with a decrease in antidiuretic hormone (ADH) and increase in atrial natriuretic peptide (ANP) (Miyamoto et al., 1991; Goldinger et al., 1992). Taken together, a decrease in USG may occur even when TBW is low – and *vice versa*.

### Strengths and limitations

The data for this study was collected during a commercial diving campaign with a minimum of changes to the divers’ routine. However, minimal interference complicates the execution of research. The divers were asked to do their BIA measurements and urine collections at set times before and after bell runs. But as other task relevant for the work, e.g., bell checks, maintenance, testing of equipment, briefs or similar had to be prioritized, there was inevitable variation in the timing of data and urine collections.

Food and drink consumption were not recorded, and neither were changes in body weight or net fluid loss. This was not central to the aim of this study but could be included in future studies to examine in detail how the divers compensate for fluid loss. A useful comparison could be made to a study by Deb et al. (2021), who did a comprehensive analysis of the energy intake and expenditure during a similar dive. This dive was done under similar conditions, and on the same DSV. Tracking the types, volumes and times of fluid intake would also be useful when interpreting the USG and BIA data to eliminate bias from intake shortly before measurements (Dixon et al., 2006; Logan-Sprenger and Spriet, 2013).

The impact of sea-water immersion on the conducting properties of human skin is not fully mapped out. BIA is heavily influenced by the conduction properties of the skin and electrodes (González-Correa and Caicedo-Eraso, 2012) and for this reason the electrode gel was used to standardize the contact surface. This does not, however, affect the properties of skin itself. Long term exposure of sea water makes the skin absorb moisture, which has been shown to increase the conductivity and thus decrease the impedance (Björklund et al., 2013). Also, the temperature of the skin effects the impedance inversely (Gudivaka et al., 1996). This was not controlled for in the study.

## Conclusion

In conclusion, the divers successfully maintained their hydration levels during the bottom phase a commercial saturation campaign with daily bell runs for underwater work. A negative correlation between BIA and USG measurements may reflect differences in what the two methods measure.

## Data Availability

The raw data supporting the conclusions of this article will be made available by the authors, without undue reservation.
